# An automatic driving trajectory planning approach in complex traffic scenarios based on integrated driver style inference and deep reinforcement learning

**DOI:** 10.1371/journal.pone.0297192

**Published:** 2024-01-25

**Authors:** Yuchen Liu, Shuzhen Diao

**Affiliations:** DEEPROUTE.AI, Columbia University, Fremont, CA, United States of America; Chunghwa Telecom Co. Ltd., TAIWAN

## Abstract

As autonomous driving technology continues to advance and gradually become a reality, ensuring the safety of autonomous driving in complex traffic scenarios has become a key focus and challenge in current research. Model-free deep reinforcement learning (Deep Reinforcement Learning) methods have been widely used for addressing motion planning problems in complex traffic scenarios, as they can implicitly learn interactions between vehicles. However, current planning methods based on deep reinforcement learning exhibit limited robustness and generalization performance. They struggle to adapt to traffic conditions beyond the training scenarios and face difficulties in handling uncertainties arising from unexpected situations. Therefore, this paper addresses the challenges presented by complex traffic scenarios, such as signal-free intersections. It does so by first utilizing the historical trajectories of adjacent vehicles observed in these scenarios. Through a Variational Auto-Encoder (VAE) based on the Gated Recurrent Unit (GRU) recurrent neural network, it extracts driver style features. These driver style features are then integrated with other state parameters and used to train a motion planning strategy within an extended reinforcement learning framework. This approach ultimately yields a more robust and interpretable mid-to-mid motion planning method. Experimental results confirm that the proposed method achieves low collision rates, high efficiency, and successful task completion in complex traffic scenarios.

## 1. Introduction

Over the past decade, significant research and practical efforts have been devoted to the development of autonomous driving technology. This development is driven by the pressing issues of increasing global vehicle ownership, leading to worsening traffic congestion and a rise in traffic accidents [1]. According to statistical data from the U.S. National Highway Traffic Safety Administration in 2021, the death rate due to traffic accidents increased by approximately 7% during the same period, partly attributed to deteriorating driving habits [2]. Human factors have contributed to frequent traffic accidents, making the deployment of autonomous driving technology a compelling solution. Autonomous driving technology utilizes high-precision sensing and efficient computational planning systems, which can significantly enhance traffic efficiency and safety during travel [3]. Consequently, in recent years, driven by policy initiatives across different nations, autonomous driving technology is advancing towards practical application and commercialization.

**Fig 1:**
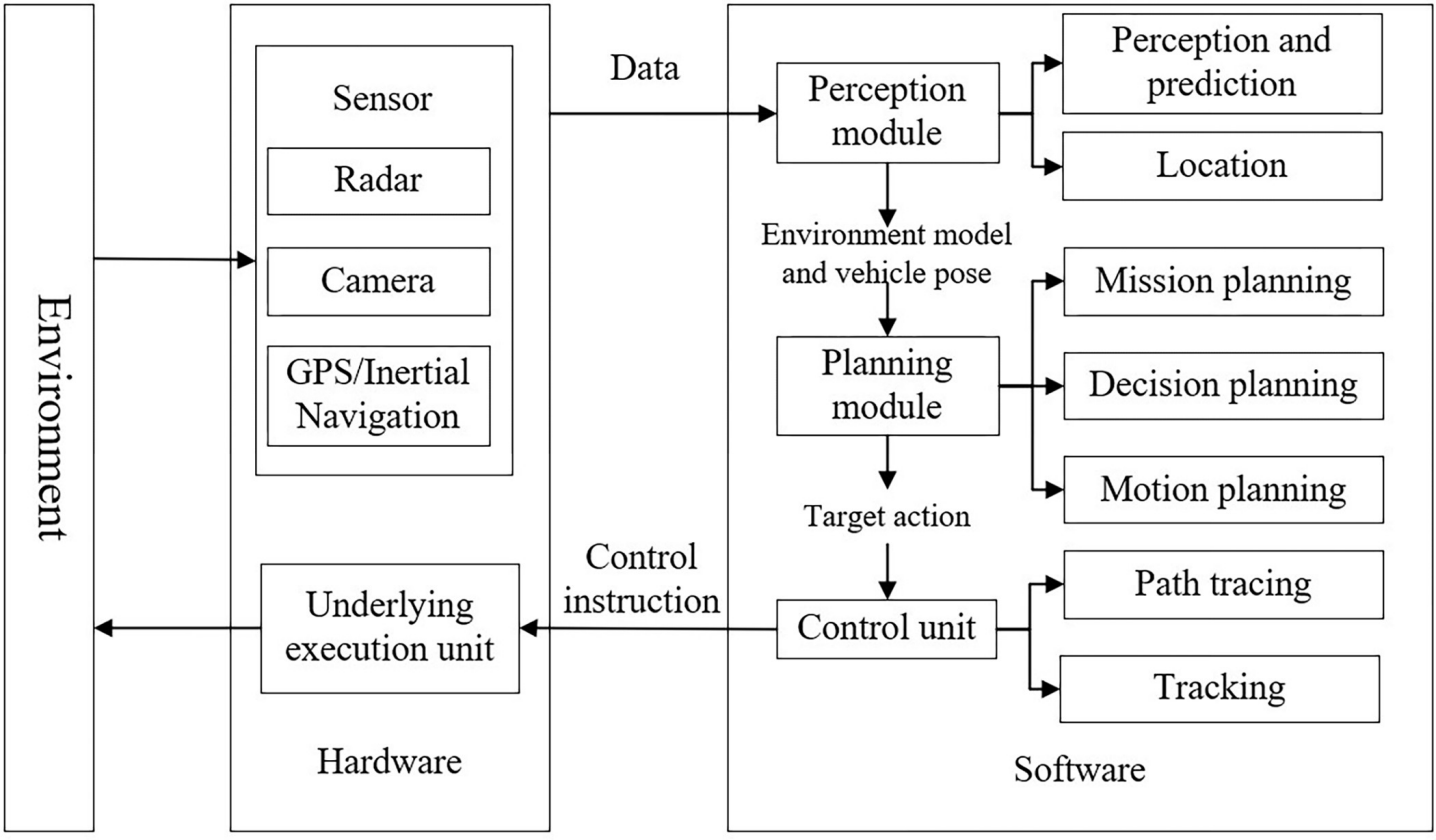
Architecture of autonomous driving system.

The core technologies of autonomous driving encompass modules such as Perception & Location, Prediction, Planning, and Control [4, 5]. These modules integrate a wide array of technologies from automatic control, artificial intelligence, multisensory information fusion, and communication systems. The system architecture is illustrated in Fig 1. Autonomous driving systems rely on perception technology to gather information about the surrounding environment, enabling vehicle localization and even predictions about the future states of other traffic participants. The Planning module processes information from the Perception and Prediction modules and operates on three levels: task planning, which maps a global path from the starting point to the destination based on waypoints, often incorporating navigation information via the Localization module; decision planning, which involves high-level driving behavior decisions; and motion planning, which, using the navigation path and driving decision, generates a feasible and safe trajectory for the vehicle, allowing it to securely follow the path to the designated location. The Control module receives instructions from the planning module and controls the vehicle’s movement [6, 7].

Planning serves as the brain of autonomous driving, occupying a pivotal role in the system hierarchy. It synthesizes information from various sources to plan the behavior and actions of autonomous vehicles. Any error in one module can directly impact behavioral decisions, subsequently affecting the entire driving process. As the research in autonomous driving technology advances, the focus has shifted from simple, controlled environments to more challenging, complex, and adverse conditions [8]. In these complex environments, autonomous vehicles encounter traffic scenarios that are challenging even for human drivers. These scenarios often include dense traffic, obstructed visibility, and various unexpected events. In a mixed traffic environment where communication may be absent, and autonomous vehicles share the road with human traffic participants, complex interactions occur. In complex traffic scenarios, uncertainties stemming from obscured vision and future behavior of other traffic participants make it difficult for autonomous vehicles to construct an accurate model of their surroundings, posing significant challenges to decision-making and planning for autonomous driving [9]. Traditional rule-based planning methods face limitations when dealing with complex scenarios. While deep reinforcement learning-based planning methods exhibit improved scalability in complex environments, they lack the ability to reason about uncertainties. Moreover, their end-to-end nature raises concerns about reliability and robustness. Overcoming these challenges to expedite the deployment of autonomous driving technology remains a key area of focus for researchers.

With the increasing integration of autonomous driving technology into open environments, autonomous vehicles are expected to operate within mixed traffic flows alongside other societal vehicles. In complex traffic scenarios, interactions between autonomous vehicles and surrounding vehicles become more frequent and intricate. Therefore, accurately predicting the latent states of other traffic participants, such as their driving styles, is crucial to facilitate improved interactions between autonomous and other vehicles. This is of significant importance in enhancing the safety and reliability of autonomous driving planning. The potential states of traffic participants encompass their driving intentions and driving styles, with driving intentions being the primary factor influencing trajectory predictions by autonomous vehicles. Planning modules can use these predictions to chart collision-free routes. In complex interactions, however, attributes with relatively persistent characteristics, specifically driving styles, become critical for negotiating between vehicles [10]. Currently, in autonomous driving research, prediction and inference mostly operate as separate modules and are not closely integrated with planning. Most studies addressing autonomous driving planning in complex traffic scenarios primarily consider collision avoidance, overlooking the influence of potential states, such as driving styles, on vehicle interactions. As a result, the underlying negotiations and bargaining processes and potential hazards in vehicle interactions often go unnoticed. Even the most advanced autonomous driving technologies currently available struggle to ensure safety when confronted with the complexity and uncertainty of real-world traffic scenarios [11].

In light of the aforementioned backdrop, to enable autonomous vehicles to safely navigate complex traffic scenarios and efficiently interact and negotiate with surrounding vehicles, this study investigates driving style prediction, autonomous driving planning, and the tight coupling of the two. To address the issue of existing planning algorithms lacking the capability to infer surrounding vehicles’ driving styles, we propose a driving style inference network based on Variational Auto-Encoder (VAE) and Gated Recurrent Unit (GRU). This network leverages historical trajectory data of nearby vehicles obtained through perception to infer their driving styles. Furthermore, we introduce a stability controller based on Lyapunov functions to enhance the robustness and stability of existing deep reinforcement learning algorithms. We also integrate driving style features with other state parameters and train the system within the deep reinforcement learning framework to obtain motion planning strategies. This equips autonomous vehicles to successfully navigate complex traffic scenarios, interact with vehicles of varying driving styles, and safely navigate challenging road conditions. The contributions of this study are summarized as follows:

- Propose a driving style inference network based on VAE+GRU. Leveraging raw historical trajectory information of surrounding vehicles obtained through perception, and construct a multidimensional dynamic scene feature map. Use a traffic information module to extract traffic constraint information and build multi-information trajectories. The encoder of the VAE+GRU network learns to distinguish different features from the constructed multi-information historical trajectories, inferring the driving styles of surrounding vehicles. The decoder, based on a mixed attention mechanism, reconstructs the inferred driving style, a latent feature, back into trajectory information, enabling deep reinforcement learning to acquire safer planning strategies.
- Introduce the Lyapunov-Based Safety Actor-Critic (LBSAC) algorithm, based on Lyapunov stability theory. To address the issue of poor robustness of existing deep reinforcement learning methods when dealing with uncertain traffic scenarios, a Lyapunov function satisfying data-based stability theorems is incorporated into the critic network as a policy gradient. This addition helps the algorithm learn stable policies and ensures that the method produces safe and reliable strategies, particularly in highly uncertain environments.
- Combine the driving style inference method with the LBSAC reinforcement learning framework, resulting in a more robust and interpretable Mid-to-Mid motion planning method called Driving Style Inference-LBSAC (DSI-LBSAC). By processing observational information and its latent states to obtain driving styles, we integrate this driving style information with state information, aiding the LBSAC algorithm in learning motion planning outcomes.

## 2. Related works

### 2.1 Autonomous driving planning methods

Presently, planning methods for autonomous driving can be broadly categorized into four main classes: rule-based methods, model-based methods, and reinforcement learning-based planning Methods [12].

1. Rule-Based Planning Methods
  Rule-based methods represent the classical decision-making approach employed in early autonomous driving research. These methods involve dividing a vehicle’s driving behavior into various categories, and establishing decision rule sets based on driving regulations, driver experience, traffic knowledge, and traffic laws. These rules are used to determine the driving behavior of autonomous vehicles based on different environmental cues [13]. After receiving driving decision commands, Chen et al. employed a Dijkstra-based method to plan feasible driving trajectories [14]. Yu et al. [15] considered constraints from surrounding vehicles during lane-changing maneuvers and utilized a third-degree polynomial to plan intelligent vehicle lane-change trajectories. Yang et al. [16] employed Bézier curves to plan smooth paths for lane switching, adjusting the maximum curvature of the path through parameter design. Yu et al. [17] proposed a method based on polynomial curves to plan trajectories and speeds for intelligent vehicle lane changes. Curve interpolation-based methods generally exhibit trackability, low computational costs, and high real-time performance. Additionally, these methods are often used in conjunction with other planning approaches for trajectory smoothing. Wu et al. [18] introduced a collaborative evolution lane-changing trajectory planning method, achieving intelligent vehicle lane changes by integrating curve interpolation methods with deep learning models.
  However, rule-based methods necessitate extensive manual design of driving strategies, making them ill-suited to complex traffic scenarios that involve the randomness of surrounding traffic participants and environmental uncertainties.
2. Model-Based Planning Methods
  Qiao et al. proposed a Hierarchical Options for MDP (HOMDP) framework to address the challenge of the vast state and action spaces in POMDP models. Unlike POMDP, HOMDP utilizes a multi-layered selection network during decision-making, assessing the trustworthiness of the vehicle’s current driving environment solely based on the current observation, without considering past observations. The HOMDP algorithm was validated in a crossroads scenario [19]. The team led by Shaojie Shen at the Hong Kong University of Science and Technology introduced a Multi-policy Decision Making (MPDM) system to handle decision-making challenges in complex traffic scenarios. They employed the Spatiotemporal Semantic Corridor (SSC) method for motion planning [20]. Subsequently, researchers proposed enhancements to the MPDM system, replacing multiple decision trees with a domain-specific closed-loop strategy tree to improve the decision system’s continuity. They also introduced a conditional focus branching mechanism to address the challenge of information overload from an excessive number of surrounding vehicles, evolving into an Efficient Uncertainty-aware Decision-making (EUDM) system [21]. Li et al. addressed the challenge of circular intersection road environments by proposing an online solution for autonomous driving decision-making in partially observable Markov decision processes, thereby enhancing computational accuracy and solution efficiency [22]. They also introduced the use of a Multi-policy Decision Making (MPDM) system to tackle decision-making challenges in complex traffic scenarios. Additionally, they employed the Spatiotemporal Semantic Corridor (SSC) method for motion planning [23].
  However, current POMDP models for autonomous driving are excessively simplistic, with overly strong assumptions during the modeling process, making them inadequate for effectively handling the substantial randomness inherent in the driving process.
3. Reinforcement Learning-Based Planning Methods
  Researchers such as Sharifzadeh utilized an inverse reinforcement learning algorithm based on Deep Q-Networks to extract reward functions from expert driving data, assisting autonomous driving in learning lane-changing behaviors more akin to human drivers [24]. Rezaee et al. explored the application of the Maximum Entropy Reinforcement Learning method in complex traffic scenarios, particularly at intersections, with a focus on addressing motion planning challenges in situations involving obstructed views. Unlike rule-based and model-based methods, reinforcement learning-based end-to-end planning methods incorporate perceptual processing into neural networks [25]. Currently, research in hierarchical planning primarily focuses on two levels: planning up to the control layer, which manages speed and steering angles, and planning up to the decision layer, which includes actions such as moving straight or turning left or right. It is essential to note that even when planning extends only to the decision layer, the tight coupling between decision-making and motion planning remains a consideration. Directly issuing control commands using reinforcement learning algorithms can introduce safety risks due to the black-box nature of the approach. Consequently, in recent years, scholars have proposed end-to-end motion planning, employing reinforcement learning to generate driving trajectories as actions while ensuring safety, thus maximizing the advantages of end-to-end methods [26]. Li et al. introduced an integrated methodology for automated lane change systems in automated vehicles, addressing limitations in existing works by combining reinforcement learning for decision making with a specially devised trajectory planning model [27]. Gu et al. developed a motion planning method for automated driving that combines reinforcement learning and Curve interpolation strategy [28]. Hu et al. addressed the AGV conflict prevention path planning by proposing a multi-agent deep method [29].

### 2.2 Planning methods based on driving style inference

Urban autonomous driving encounters a multitude of complex traffic scenarios, such as dense traffic flow, signal-free intersections, and uncertainty caused by occlusions, all of which intensify the complexity of vehicle interactions, subsequently impacting driving safety. To address this challenge, many researchers have integrated inferred driving styles of surrounding vehicles based on perceptual information, including historical states and trajectories, directly into reinforcement learning algorithms during the study of autonomous driving decision planning.

Ma and colleagues employed a supervised learning approach to categorize and label driving styles. They represented intersection scenarios as undirected graphs and employed graph neural networks to infer the driving styles of surrounding vehicles and their interactions, aiding reinforcement learning in decision planning [30]. Inferring driving styles from historical trajectories is a quintessential sequence data representation learning task. Variational Autoencoders (VAEs) have demonstrated notable performance in representation learning studies, and various research efforts currently utilize VAEs and their variants to process autonomous driving perception data [31]. Consequently, some scholars have integrated VAEs into the tight coupling of driving style inference and reinforcement learning-based planning. For instance, Morton and colleagues employed a VAE network structure based on Recurrent Neural Networks (RNNs) to encode the driving trajectories of human-driven vehicles with distinct driving styles into a latent space. This latent encoding, along with the current driver’s state, is fed back into a feedforward policy that produced multimodal actions, achieving joint optimization of the encoder and driving strategy [32]. Liu and others addressed T-shaped signal-free intersections and employed a Variational Autoencoder with a recursive neural network to learn the latent representation of features that lacked fundamental ground truth labels. Subsequently, they used this feature representation with a deep reinforcement learning algorithm to learn planning strategies, enabling autonomous vehicles to safely negotiate interactions with other vehicles in this scenario and enhance efficiency at intersections [33]. Wang et al. presented a trajectory planning approach for self-driving vehicles in uncertain intersection scenarios, the proposed approach is designed on reinforcement learning and Transformer [34]. Liu et al. proposed conditional variational autoencoder to generate the vehicle trajectory [35]. However, supervised learning necessitates substantial labeling, and the cost of acquiring feature labels is often prohibitively high and typically absent from most real driving datasets [33]. Current unsupervised learning-based joint optimization methods are limited to specific driving scenarios, and their network structures may not effectively extract implicit traffic constraint relationships among vehicles during the driving process. Consequently, planning for autonomous driving in complex traffic scenarios remains an unresolved challenge that warrants further exploration, particularly in the context of incorporating the inferred driving styles of surrounding vehicles.

## 3. The proposed method

### 3.1 Planning modeling based on POMDP

In complex traffic scenarios, there exist numerous pieces of information that autonomous driving systems cannot observe. The uncertainty arising from unobservable factors presents a significant challenge that needs to be addressed in the planning process for autonomous driving. Therefore, autonomous driving planning falls into the category of Partially Observable Markov Decision Process (POMDP) problems. In this paper, we model the planning problem for autonomous driving in complex traffic scenarios as a POMDP.

#### 3.1.1 Foundations of POMDP

The Markov Decision Process (MDP) describes a sequential decision-making process where an agent interacts with the environment. In cases of partially observable Markov decision processes (POMDP), the ideal model for sequential decision-making is considered when the environmental state is only partially known due to dynamic uncertainties. The MDP, represented by the tuple 〈*S*,*A*,*T*,*R*,*y*〉, which can be transformed into a POMDP by introducing an observation function Ω:*S*→*O* when the state is not directly observable. The relationship between the state *s*∈*S* and the observation *o*∈*O* is defined by function Ω, within the observation space *O*. Hence, the POMDP tuple can be expressed as 〈*S*,*A*,*T*,*R*,Ω,*O*,*y*〉, and ρ_0_ denotes the initial state distribution. The state transition probability function $T(s,a,s^{'})=p(s^{'}|s,a)$ dictates the probability distribution of the new state *s*’ when action *a* is taken in the current state *s*. Analogous to MDPs, solving POMDP problems involves finding a policy π:*O*→*A* that maximizes the expected cumulative reward:

$$\pi^{*}=\underset{\pi }{\;\arg\;\max}\;E_{s_{0},a_{0},o_{0},\cdots }\sum_{t=t_{0}}^{\infty }y^{t}R(s_{t},a_{t})$$ (1)

where $s_{0}∼\rho_{0}(s_{0})$, $a_{t}∼\pi (a_{t}|o_{1:t})$, $o_{t}∼\Omega (o_{t}|s_{t})$ and $s_{t+1}∼T(s_{t+1}|s_{t},a_{t})$.

Given the characteristics of POMDP and the dynamic uncertainty and partial observability inherent in autonomous driving scenarios, this paper employs the POMDP model to formulate the planning and decision-making problem.

#### 3.1.2 Observation model

In single-agent autonomous driving (ego-vehicle), environmental information primarily consists of the state information of surrounding human-driven vehicles and the ego-vehicle’s own state within the traffic road environment. The number of surrounding vehicles *n* may change over time *t*. All vehicles are modeled as moving in Frenet coordinate space. $o_{0}^{t}$ represents the state of the ego-vehicle at time *t*, and $o_{i}^{t}$ represents the state of the *i*_*th*_ surrounding vehicle at time *t*, *i*∈{1,2,⋯,*n*}. The state information includes longitudinal displacement *s*, lateral displacement *d*, and speed (*v*_*s*_,*v*_*d*_). These features reflect the interaction between vehicles during the driving process. Additionally, the acceleration information of human-driven vehicles to some extent reflects their intentions at specific moments. Existing methods often overlook the influence of intentions on driving styles, which are persistent attributes. Changes in intent can lead to abrupt style shifts, making the inferred driving styles less precise in complex traffic scenarios. Therefore, it is necessary to extract acceleration information from the state information of surrounding vehicles. Based on the current state of computer vision algorithms, precise location information, after noise reduction, can be obtained from the perception module, while speed and acceleration can be approximated from the first and second derivatives of displacement, with Δ*t* representing the adjacent time intervals.


$$\begin{array}{l} v_{s}^{t}={\dot{s}}^{t}=\frac{s^{t}-s^{t-1}}{\Delta t}\\ v_{d}^{t}={\dot{d}}^{t}=\frac{d^{t}-d^{t-1}}{\Delta t}\\ a_{s}^{t}={\ddot{s}}^{t}={\dot{v}}_{s}^{t}=\frac{v_{s}^{t}-v_{s}^{t-1}}{\Delta t}\\ a_{d}^{t}={\ddot{d}}^{t}={\dot{v}}_{d}^{t}=\frac{v_{d}^{t}-v_{d}^{t-1}}{\Delta t} \end{array}$$ (2)

**Fig 2:**
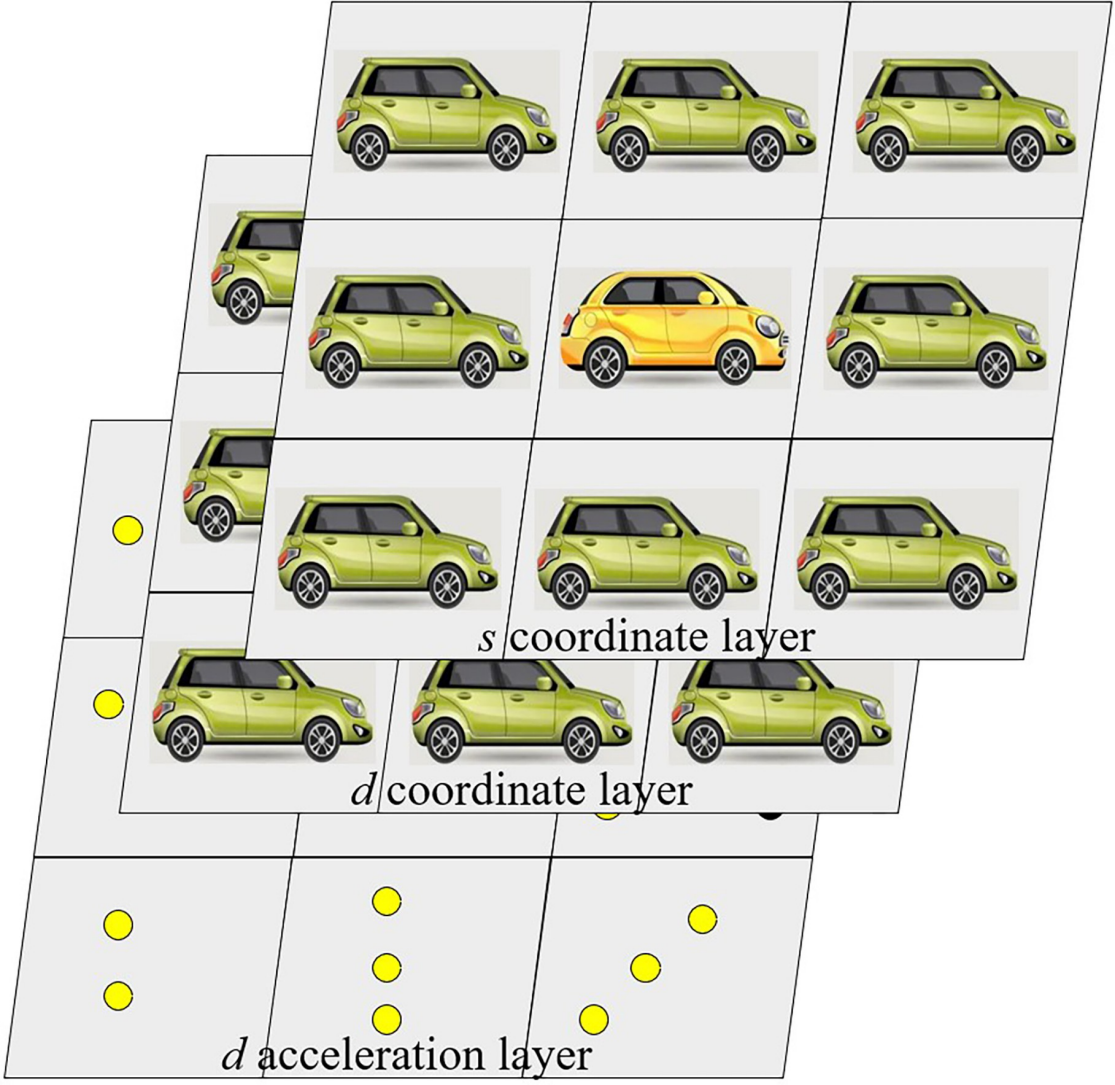
Multidimensional dynamic scene feature map observed under the POMDP model.

Utilizing the multidimensional feature information obtained through sensors and basic processing, a dynamic feature map of autonomous driving is constructed. Centered on the ego-vehicle, a 3×3 rectangular grid, comprising nine cells, is created to map surrounding vehicles according to their relative positions. Position layers, velocity layers, and acceleration layers are individually formed for *s* and *d* coordinates. These layers together create a 6×3×3 multidimensional dynamic scene feature map, as illustrated in **Fig 2**.

In complex traffic environments, the aforementioned state information is only partially observable. Increased uncertainties arise in scenarios with occlusions or dense traffic, rendering the state information of surrounding vehicles incompletely observable. In cases of occlusion, it may be impossible to perceive even the positional information of certain vehicles, rendering them entirely unobservable. Consequently, the feature map contains missing information, and the interaction relationships need to be further learned through reinforcement learning methods presented in subsequent sections. Moreover, each surrounding vehicle has a latent state *z*_*i*_∈{*conservative*,*aggressive*} representing the driving style of the *i*_*th*_ driver. This latent feature encapsulates the conservative and aggressive attributes of driving styles. Unlike other state information, driving style cannot be directly observed or easily calculated.

### 3.2 Extraction of vehicle trajectories and representation of state information

#### 3.2.1 Vehicle trajectory information extraction network

**Fig 3:**
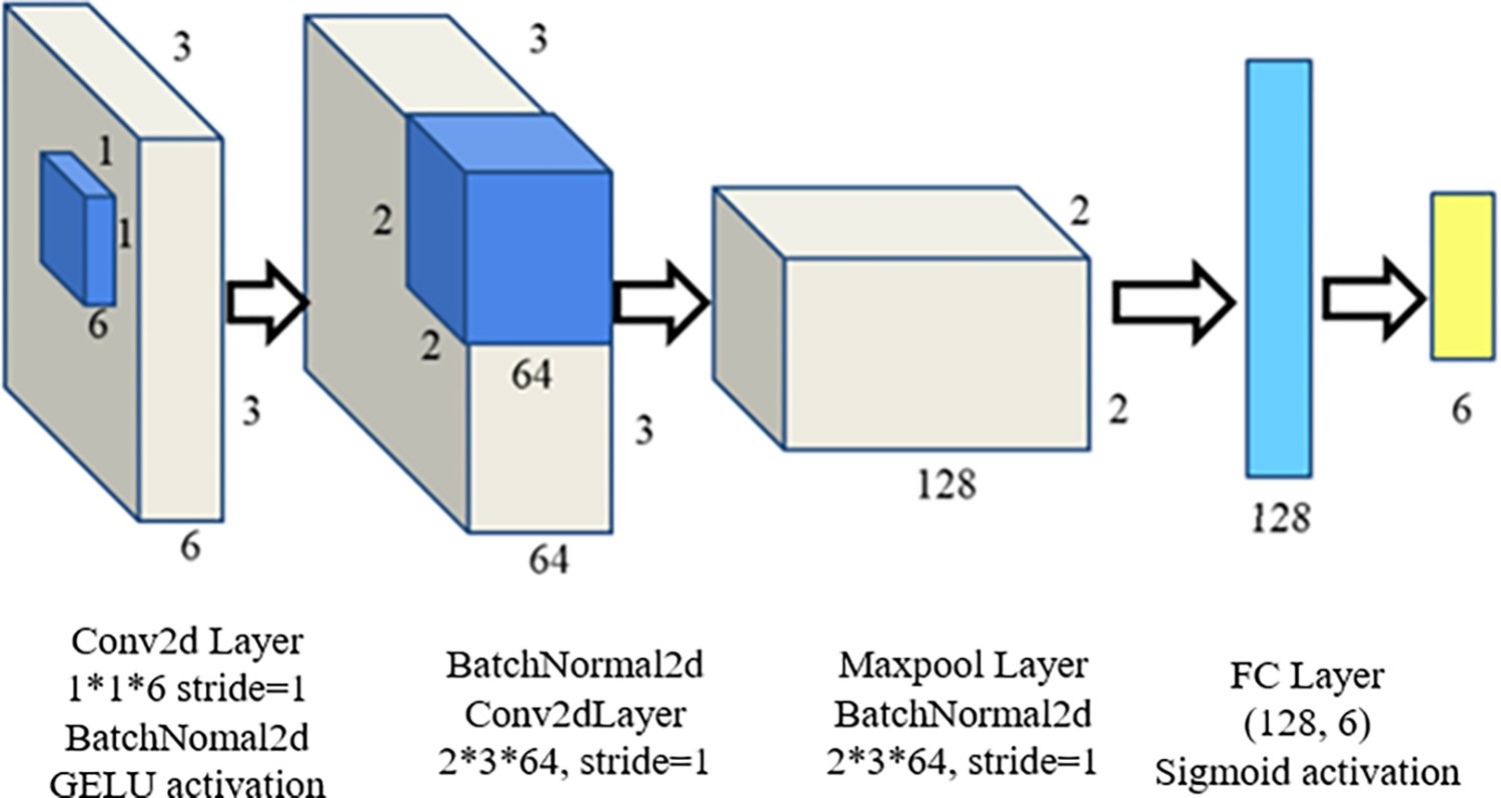
Traffic information extraction network.

The multi-dimensional dynamic feature map constructed from perceptual observation data reflects all observed information within the model. However, the implicit interactions between surrounding traffic participants cannot be directly discerned from position and velocity information. In the past, information such as position, velocity, and acceleration within the multi-dimensional dynamic scene feature map encapsulated intentions, implicit interactions, and constraints of surrounding vehicles—details that were absent in conventional trajectory data consisting solely of positional points. This paper introduces a network designed to process the multi-dimensional dynamic scene feature map and acquire multi-modal trajectory data enriched with pertinent traffic information. As illustrated in Fig 3, a 1×1 convolution kernel is employed to fuse the multi-dimensional features of surrounding human drivers. Subsequently, a 2×2 convolution kernel is used to enable interactions between the autonomous vehicle and surrounding vehicles, thus extracting the interactions among vehicles. Finally, information *q*, reflecting the interactions and constraints between vehicles, which underlie the trajectories, is obtained through Maxpool and FC layers. This information enriches the trajectory data with multi-modal insights.

#### 3.2.2 Representation of vehicle trajectories and state information

Inference of latent state information, such as the driving styles of surrounding vehicles, must be deduced from the historical trajectory data containing position and corresponding velocity information. Essentially, inferring latent driving styles equates to feature representation learning. Historical trajectory data of vehicles, consisting of positional points and corresponding velocity information, can be represented as $x_{i}^{t}$. The distribution of actions for the *i*_*th*_ human driver is modeled as $P(A_{i}^{t}|x_{i}^{t},z_{i}^{t})$. The aim of latent state inference is to learn $P(z_{i}^{t}|x_{i}^{1:t})$, where $x_{i}^{1:t}$ denotes the historical observed trajectories of surrounding vehicles from the initial time to time *t*. To facilitate the learning of feature representations for trajectories and latent state inference, trajectory data for crossroad scenarios is needed. Prior to collecting trajectory information in complex traffic scenarios, an elucidation of trajectory observations for crossroad environments is essential.

**Fig 4:**
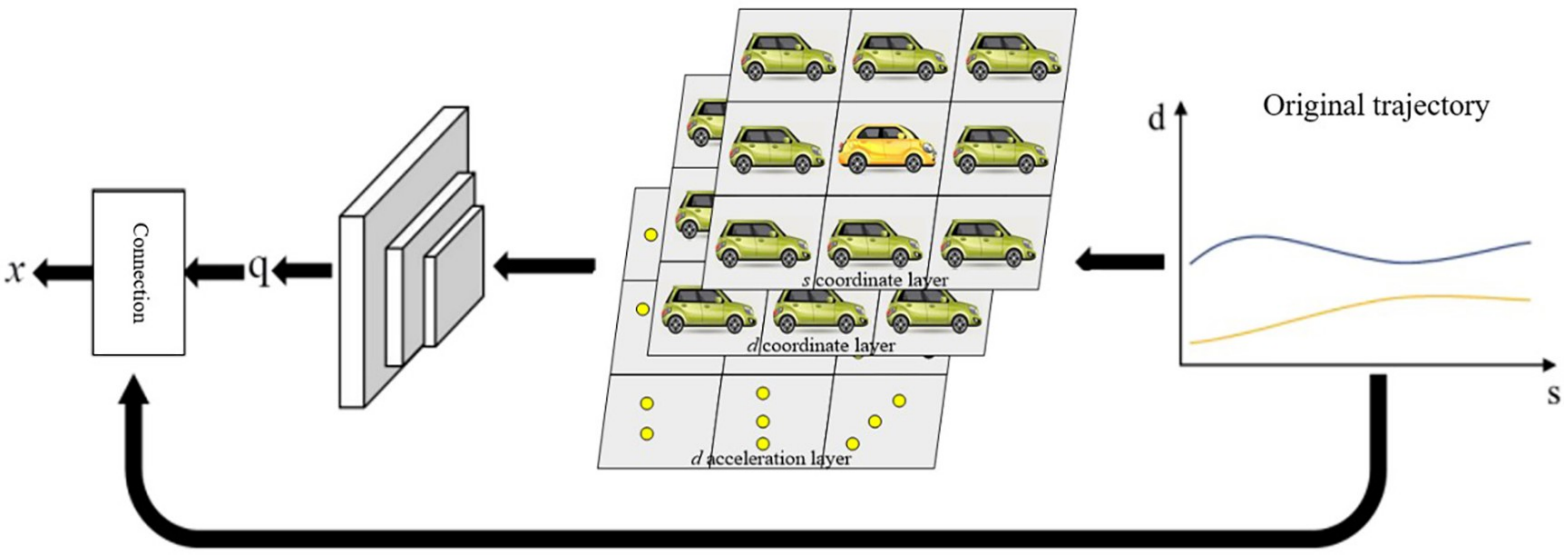
Traffic observation information processing process.

Traffic observation information processing process is llustrated in Fig 4. During the driving process, with time *t* as the unit of measurement, each vehicle’s trajectory can be represented by its positional information at each time step, with the density of adjacent trajectory points reflecting speed information. Therefore, during the feature representation learning phase, each vehicle’s observable state, namely trajectory data, is simplified to *x* = (*s*,*d*|*q*). *s* represents the longitudinal displacement of the vehicle from its initial state, *d* represents the lateral displacement, and *q* incorporates the interaction constraints information extracted through the multi-dimensional dynamic features and traffic information module. The trajectory state information for each driver can be represented as τ = [x^1^,⋯,*x*^*t*^]. Considering the variability of vehicle conditions in complex traffic scenarios, the past 20-time steps are selected as a segment of trajectory. The collected trajectories are rotated to align all trajectories in the dataset in the same direction. Consequently, lane and direction information is indistinguishable in trajectory data, enabling the network to focus on learning latent feature differences rather than other disparities among vehicles.

Simulations were conducted for traffic scenarios without autonomous vehicles at signalized crossroads, recording the trajectories of all surrounding vehicles controlled by the Intelligent Driver Model (IDM) [36]. By learning feature representations from this dataset, autonomous vehicles can infer features from other drivers’ trajectories before deciding to merge or wait. The trajectory dataset is denoted as ${\left\{\tau_{j}\right\}}_{j=1}^{N}$, where *N* represents the total number of trajectories.

### 3.3 Inference of driving style

**Fig 5:**
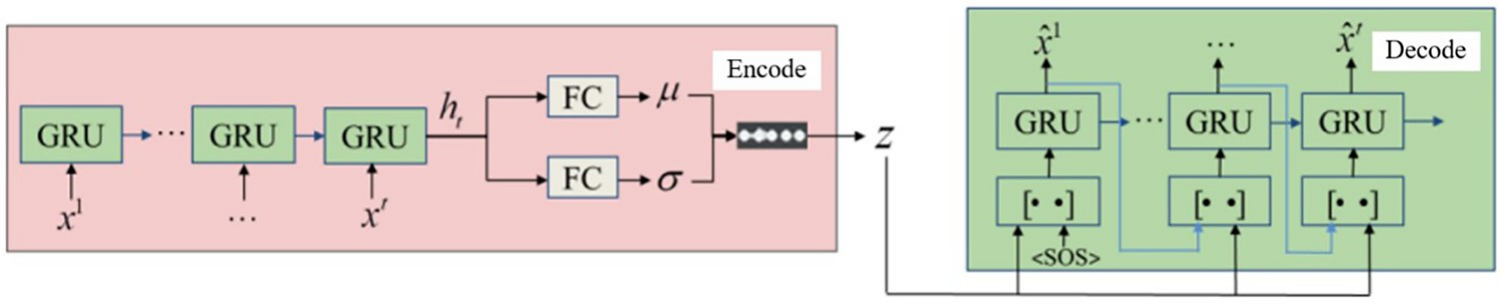
GRU+VAE latent state inference network architecture.

Utilizing a VAE+GRU network structure for encoding and decoding the trajectories of surrounding vehicles, we learn the feature representations within the trajectories and infer driving styles. The network architecture of this method is depicted in **Fig 5**. In the diagram, [••] represents information concatenation. The VAE network consists of an encoder and a decoder, with the former compressing the trajectory τ into a distribution of latent feature vectors *z*, while the latter utilizes these latent feature vectors to reconstruct the trajectory.

#### 3.3.1 VAE representation learning based on RNN

By employing an RNN to refine and enhance the VAE, we create an autoencoder for data sequences with a Gaussian prior acting as a regularization term for the hidden encoding. The decoder is a specialized RNN inference model that conditions on the hidden encoding and contains no useful information in a degenerate environment. LSTM networks, due to their long-term memory capabilities, are commonly used in fields such as classification and prediction. The VAE+RNN representation learning model quantifies the degree to which the model learns global features by observing the variational lower bound. By encoding useful information in the latent variable *z*, the model has a non-zero KL divergence term and relatively small cross-entropy term, which cannot be achieved directly using VAE alone. The LSTM decoder captures subtle changes in the hidden states, requiring the use of global latent variables to achieve good likelihood.

Applying the VAE+RNN representation learning method to driving style inference, we learn the features from the historical state information of surrounding vehicles and infer the corresponding driving styles. Since the potential driving styles of other vehicles affect the autonomous driving strategy, maximizing the objective needs to consider this latent state.


$$\begin{array}{l} \log(a_{t}|s_{t})=\log\int p(a_{t},z|s_{t})dz\\ \;=\log\int p(a_{t}|z,s_{t})p(z|s_{t})dz \end{array}$$ (3)

To address the issue of unknown latent states, this study encodes the driving trajectories of different driving styles into a latent space to obtain a cognitive model *q*(*z*|*a*,*s*) closely approximating the true posterior distribution. Subsequently, the latent encoding and the current driver’s state are fed into a feedforward policy to produce multimodal actions, transforming Eq (3) as follows:

$$\begin{array}{l} \log(a_{t}|s_{t})=\log\int p(a_{t}|z,s_{t})p(z)\frac{q(z|a,s)}{q(z|a,s)}dz\\ \;=\log E\left[\frac{p(a_{t}|z,s_{t})p(z)}{q(z|a,s)}\right] \end{array}$$ (4)

Since the feedforward policy only considers the relationship between the current state and action, joint optimization encourages the encoder to encode short-term information about trajectories, such as acceleration. Therefore, driving style inference is not precise enough. To address this, the framework incorporates a decoder based on the GRU network for trajectory reconstruction, compelling the encoder to encode feature information.

#### 3.3.2 VAE representation learning based on GRU

Compared to LSTM, GRU has fewer parameters, faster training, and requires less data for generalization. In long-term prediction inference in complex environments, GRU outperforms LSTM [37]. Therefore, the proposed approach adopts an encoding structure based on GRU+VAE.

VAE learns a cognitive model *q*(*z*|*x*) regarding the posterior of the latent state encoding *z* through data processing, forming an ellipsoidal region in latent space where data with similar semantic features are clustered together. The autoencoder aims to train by attempting to make the reconstructed output identical to the input in terms of feature representation. As shown in **Fig 5**, the proposed VAE network comprises an encoder and a decoder. A dataset of trajectory state information is used to train the VAE+GRU network to learn feature representations. In the encoding phase, given trajectory τ = [*x*^1^,⋯,*x*^*t*^], the encoder GRU first applies a non-linear embedding layer to each state, and then provides the embedded features to the GRU units:

$$h_{e}^{t}=GRU(h_{e}^{t-1},f_{encoder}(\tau^{t}))$$ (5)

where $h_{e}^{t}$ represents the hidden states of the encoder GRU from 1 to t time steps. After the entire trajectory passes through the encoder GRU, the final hidden state is treated as the encoded latent feature of trajectory "e," and Gaussian parameters for latent driving styles τ are obtained using a fully connected layer:

$$\mu =f_{\mu }(h_{e}^{t}),\;\sigma_{i}=f_{\sigma }(h_{e}^{t})$$ (6)

Finally, the reparameterization trick is employed to sample *z* from *N*(μ,σ) for more efficient learning z=μ+εσ,ε∼N(0,I):

#### 3.3.3 Hybrid attention mechanism

As a time series inference task, the inference of driving styles in complex traffic scenarios possesses particular features. The features influencing model performance change over time. For instance, during normal driving, vehicle motion remains consistent, and driving styles are primarily inferred from historical positional and velocity information. However, when unexpected situations arise at intersections, interactions between vehicles change rapidly. In such cases, to avoid collisions, vehicles’ motion patterns change drastically (e.g., hard braking or aggressive acceleration), and driving styles might differ from previous moments. Therefore, relying solely on either time-based or feature-based attention mechanisms is insufficient to enhance the accuracy of inferring latent driving styles in complex scenarios.

To address the rapid changes over relatively short time intervals, this study introduces a hybrid attention mechanism that combines time-based and feature-based attention. This attention mechanism assesses the influence on output accuracy for each moment and each feature independently, distributing attention weights. With a finer-grained attention allocation mechanism, the model can focus on critical moments and corresponding features to produce more accurate outputs. The principles of the hybrid attention mechanism are outlined below. **Eq (7)** represents the hidden states *H* of GRU units over the past *t*_*h*_ frames:

$$H={\left[h^{t-(t_{h}-1)},\cdots ,h^{t-2},h^{t-1}\right]}^{T}$$ (7)

Assuming the hidden states are *n*-dimensional, then $H\in {\mathbb{R}}^{t_{h}\times n}$. If the hidden state at time *t* is *h*^*t*^∈ℝ^1×*n*^, the cosine similarity between *g*_*t*_ and *g*_*f*_ can be calculated for both the time dimension and the feature dimension using the scoring functions *H* and *h*^*t*^, respectively:

$$\begin{array}{l} g_{t}(H,h^{t})=Hh^{T}\\ g_{f}(H,h^{t})=h^{t}(W_{f}H) \end{array}$$ (8)

where $W_{f}\in {\mathbb{R}}^{t_{h}\times n}$. The time attention vector $a_{t}\in {\mathbb{R}}^{t_{h}\times 1}$ and the feature attention vector *a*_*f*_∈ℝ^1×*n*^ are obtained via softmax functions:

$$\begin{array}{l} a_{t}=\mathrm{softmax}(g_{t}(H,h^{t}))\\ a_{f}=\mathrm{softmax}(g_{f}(H,h^{t})) \end{array}$$ (9)

**Fig 6:**
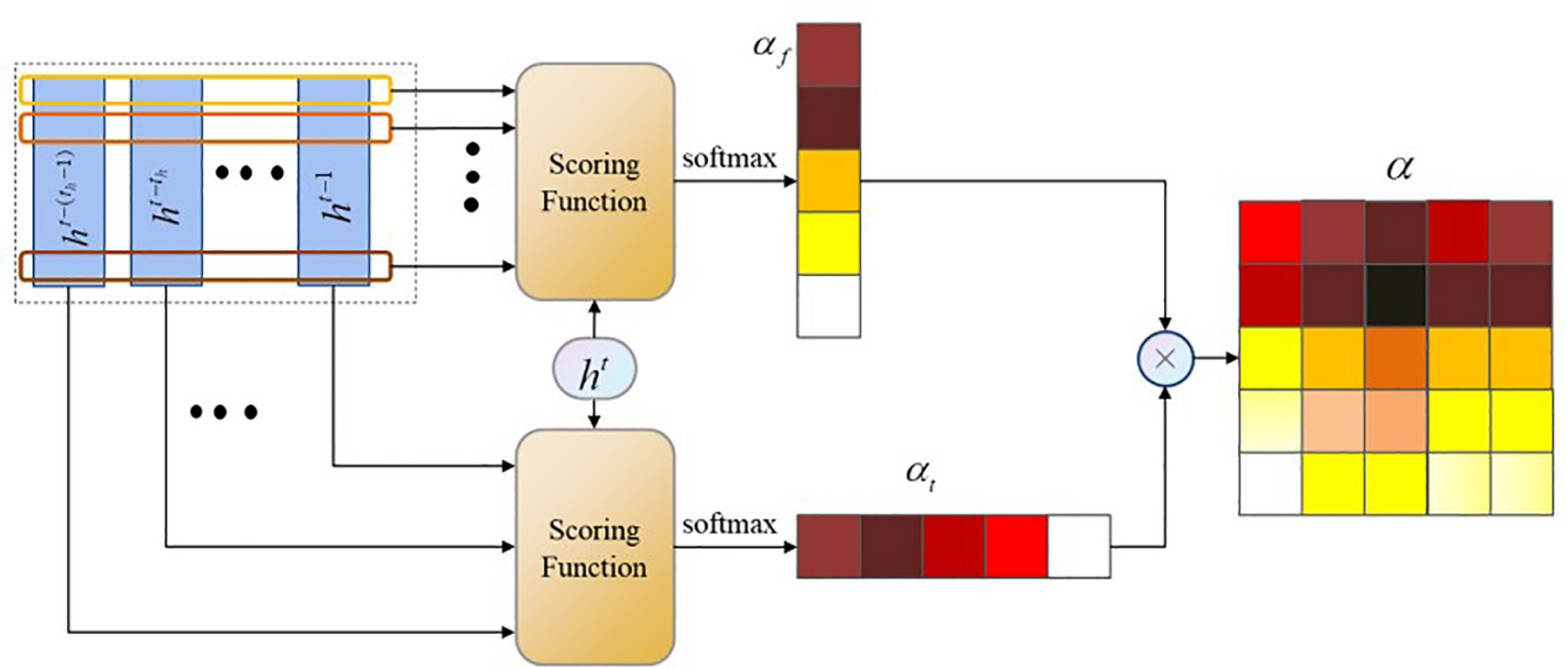
Hybrid attention module.

As shown in **Fig 6**, the final hybrid attention matrix $a\in {\mathbb{R}}^{t_{h}\times n}$ is obtained by combining both attention mechanisms:

$$a=a_{t}a_{f}$$ (10)

#### 3.3.4 Decoder based on hybrid attention mechanism

The matrix obtained by the hybrid attention mechanism assigns weights to the hidden states *H* and maximizes time and feature dimensions, selecting the moments and features most relevant to the output. During the decoding phase, since the driving style does not change within a single time step, latent state *z* is treated as a part of the surrounding vehicle state, rather than the initial hidden state of the decoder GRU. Therefore, at each time step *t*, the previous time step’s reconstructed state ${\hat{x}}^{t-1}$ is connected with the latent state *z* from the encoder. The embedded joint state is then fed into the next decoder GRU unit, where another embedding is applied to obtain the next hidden state ${\dot{h}}_{d}^{t}$ and output the next reconstructed state ${\hat{x}}^{t}$:

$$\begin{array}{l} h_{d}^{t}=contact(h_{d}^{t-1},m_{t},m_{f})\\ {\dot{h}}_{d}^{t}=GRU(h_{d}^{t},f_{encoder}({\hat{x}}^{t-1},z))\\ {\hat{x}}^{t}=g_{decoder}({\dot{h}}_{d}^{t}) \end{array}$$ (11)

In the first time step, a special sequence start state (SOS) is employed to reconstruct ${\hat{x}}^{1}$, similar to a sequence start symbol in natural language processing, until the entire trajectory $\tau =[{\hat{x}}^{1},\cdots ,{\hat{x}}^{t}]$ is reconstructed. The objective of training the VAE+GRU network is:

$$L=‐\beta D_{KL}(N(\mu ,\sigma )\|N(0,I))+{\left\|\tau -\hat{\tau }\right\|}_{2}$$ (12)

where *D*_*KL*_ is the KL divergence. The first term regularizes the distribution of the latent shape towards a standard normal distribution to make it closer to the prior. The second term is the reconstruction loss, measuring the L2-norm error between the reconstructed trajectory and the original trajectory. These two terms are weighted by *β*. By optimizing the above equation, the proposed network learns the latent encoding representing the features of each trajectory, which is the driving style, without the need for any ground truth feature labels. Furthermore, the proposed method does not assume the number of feature classes or the semantics of feature classes, making it capable of handling trajectories with more complex features.

### 3.4 Robust model-free deep reinforcement learning

Existing deep reinforcement learning methods suffer from the issue of excessive parameterization, limiting the applicability of trained policies to complex traffic scenarios in autonomous driving due to uncertainties. In response to this, the Actor-Critic reinforcement learning algorithm is enhanced in this paper, introducing a Reinforcement Learning algorithm based on Lyapunov’s safety Actor-Critic approach.

With the development of deep learning, researchers have introduced numerous deep reinforcement learning methods based on the Actor-Critic framework in recent years, successfully applied in autonomous driving planning. Among them, the Deep Deterministic Policy Gradient (DDPG) algorithm and Soft-Actor-Critic (SAC) algorithm have performed exceptionally well. Within the SAC algorithm framework, a Lyapunov function is used as a critic in the policy gradient formula, introducing a Lyapunov critic that satisfies $L_{c}(s)=E_{a∼\pi }L_{c}(s,a)$. The policy objective for strategy *π* becomes:

$$\begin{array}{l} J(s,a,w,c,s^{'})=E_{\left(s,a,w,R,s^{'}\right)}[\beta \log(\pi_{\theta }(f_{\theta }(\varepsilon ,s)|s))-Q(s,f_{\theta }(\varepsilon ,s))]\\ \;+\lambda E_{v,\pi ,T_{\pi }}\Delta L(s,a,w,c,s^{'})+\nu E_{T(s|\rho ,\pi ,t_{0}),T(t_{0}|\rho ,\pi ),\pi }(L_{c}(s,a)-k_{2}) \end{array}$$ (13)

where *π*_*θ*_ is parameterized by neural network *f*_*θ*_, *ε* is an input vector consisting of Gaussian noise, *λ* is a positive Lagrange multiplier adjusted via policy gradient to control policy entropy. *D* = (*s*,*a*,*w*,*R*,*s*’) represents the replay buffer. The expression for Δ*L* is as follows:

$$\Delta L(s,a,w,c,s^{\prime })=L_{c}(s^{\prime },f_{\theta }(\varepsilon ,s^{\prime }))-L_{c}(s,a)+(\alpha_{3}+1)c-\eta^{2}\|w{\|}_{2}$$ (14)

In this proposed framework, the Lyapunov candidate function serves as a supervisory signal during training, *L*_*c*_ is updated to be an approximate value of the objective function *L*_target_ related to the chosen Lyapunov candidate. The Lyapunov function is updated using a least squares method to minimize the target function:

$$J(L_{c})=E_{D}[\frac{1}{2}{\left(L_{c}(s,a)-L_{\mathrm{target}}(s,a)\right)}^{2}]$$ (15)

The resulting algorithm is called the Maximum Entropy Deep Reinforcement Learning based on Lyapunov, named Lyapunov-Based Safety Actor-Critic (LBSAC). Utilizing this algorithm ensures that the planning policies learned by autonomous vehicles are stable and robust.

### 3.5 Motion planning based on LBSAC

#### 3.5.1 Mid-to-Mid motion planning

**Fig 7:**
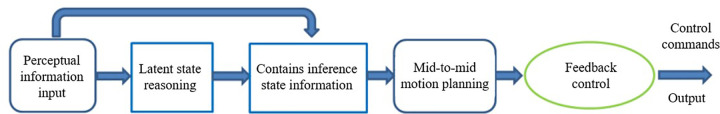
Framework diagram of the planning method in this thesis.

Prior research on motion planning using deep reinforcement learning algorithms mainly focused on an end-to-end control framework. However, this approach lacks interpretability and can lead to dangerous situations when applied in practice. Hierarchical reinforcement learning methods, which employ two agents to separately learn decision-making and motion planning layers, introduce complexity by combining discrete reinforcement learning actions with continuous observation spaces. Moreover, the sequential coupling of reinforcement agents complicates computation and destabilizes the training process because both agents need to balance exploration and exploitation. Therefore, when addressing motion planning problems in complex traffic scenarios using deep reinforcement learning, a modified framework is introduced. It processes the perceived state information to infer driving styles, incorporates this latent state into the state information, and learns the mapping from state information to motion planning-related actions using a reinforcement learning algorithm. Only the decision-making and motion planning modules are combined, while the control module tracks the motion planning output trajectory to drive the vehicle. This design retains the control module, enabling the perception module to directly send commands to the vehicle control in emergency situations, ensuring safety. This planning framework is called Mid-to-Mid, as shown in Fig 7.

#### 3.5.2 Motion planning action space design

In complex traffic scenarios, traditional pipeline-based planning methods struggle to handle environmental randomness and uncertainty. To address these issues, a deep reinforcement learning agent is designed to extract useful features from observation state information using a deep neural network and provide the optimal trajectory in a continuous action space. Training an agent under the Mid-to-Mid framework is a sensible choice. The agent processes the preprocessed observation state information and optimizes a motion planning strategy, generating a continuous polynomial trajectory in Frenet space. This approach does not generate driving commands (*θ*,*T*) based on observation information but instead produces a continuous trajectory τ*. Therefore, the key to modeling motion planning based on deep reinforcement learning lies in the processing of the state space and the determination of the action space.

**Fig 8:**
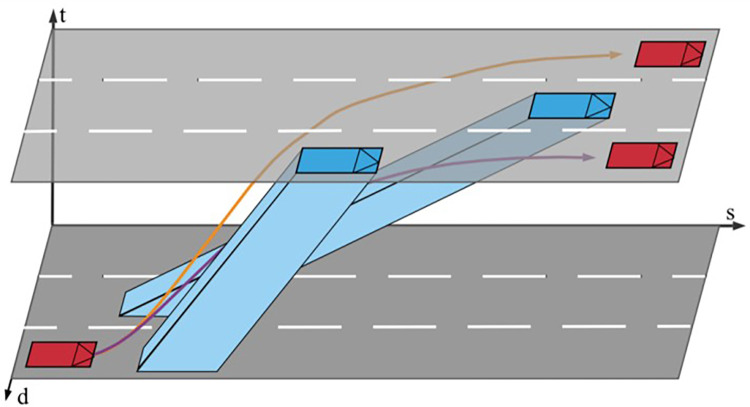
Trajectories for moving obstacles and candidate paths visualized in driving corridors.

1. State Space
  The Frenet framework is used to represent input states for the neural network, including longitudinal displacement *s* and lateral displacement *d*. Before being fed into the network, these values are normalized and transformed. The longitudinal and lateral displacements in the Frenet framework, *s* and *d*, describe the state of the autonomous vehicle and surrounding vehicles. According to the driving style inference module, the output *z* corresponding to the driving style of the surrounding vehicle is decoded and concatenated with the surrounding vehicle information. The state information *s* includes the state of the autonomous vehicle itself and the surrounding vehicle information, with the feature extraction for the information from surrounding vehicle trajectories still relying on a traffic information module network.
2. Action Space
  An agent is introduced to learn and optimize a strategy to produce a feasible trajectory in Frenet space at each time step, instead of discretizing terminal manifolds as in traditional methods. A polynomial trajectory, also known as a lattice, is represented with three continuous values: *v*_*f*_,*d*_*f*_, and *t*_*f*_. Each value has an acceptable action sampling range. By mapping these ranges to [–1, 1], a continuous action space for reinforcement learning can be defined as follows:
$$A=\{v_{f},d_{f},t_{f}\}$$ (16)
  where each value is within the range [–1, 1]. Exploring different regions of the action space is equivalent to examining different polynomial trajectories in the driving corridor, as shown in **Fig 8**. The final output of motion planning consists of feasible trajectory points composed of actions, which the control module uses to track the trajectory, thereby driving the autonomous vehicle.

#### 3.5.3 Reward function design

In terms of safety, collisions or deviations from the road pose a danger; hence, Eq (17) represents the safety reward *R*_*safe*_.


$$R_{safe}=\{\begin{array}{l} -10,\;\mathrm{if}\;\mathrm{collision}\\ 10,\;\mathrm{if}\;\mathrm{out}\;\mathrm{off}\;\mathrm{road} \end{array}$$ (17)

Regarding velocity, the speed values are designed to fall within the range between the lowest and highest speed limits, ensuring safety. In terms of driving efficiency, the vehicle receives a reward *R*_*m*_ at each time step, encouraging the vehicle to overtake slower cars, as shown in Eq (18):

$$R_{m}=10\exp\frac{-{\left(V_{\mathrm{target}}-V_{ego}\right)}^{2}}{5\times V_{\max}}$$ (18)

To encourage the vehicle to switch to a faster lane as soon as possible after overtaking slower vehicles, a lane-change reward *R*_*lc*_ is introduced. It’s important to note that lane changes resulting in a speed increase have a positive impact on the overall reward function. Frequent lane changes that do not lead to higher speeds not only decrease driving comfort but also violate traffic norms. Therefore, this paper considers the rewards of lane changes from both positive and negative aspects, with the reward function as in Eq (19):

$$\{\begin{array}{c} R_{lc}=R_{m}+r_{m}\times W_{lc+}\;\mathrm{if}\;\mathrm{speed}\;\mathrm{gain}>1m/s\\ R_{lc}=-R_{m}W_{lc-}\;\mathrm{otherwise} \end{array}$$ (19)

As defined in the equation above, the speed gain threshold is set to 1 m/s. If a lane change leads to a speed gain of at least 1 m/s, a positive lane change reward is given; otherwise, the lane change action is penalized. Here, *W*_*lc*+_ and *W*_*lc*−_ are set to 0.7 and 0.2, respectively. In summary, the reward function *R* can be represented as the sum of the above components.


$$R=R_{safe}+R_{m}+R_{lc}$$ (20)

## 4. Algorithm validation and simulation analysis

Urban autonomous driving encounters a multitude of complex traffic scenarios due to the uncertainty of the driving environment. These scenarios include situations with obstructions, heavy traffic, uncoordinated traffic signals, and obstructed paths due to broken-down vehicles. Collecting real-world data on actual roads is challenging, and training autonomous vehicles based on deep reinforcement learning in real traffic conditions can pose safety risks. Therefore, to assess the feasibility, safety, and stability of the proposed planning method in addressing typical complex traffic scenarios, this paper leverages the open-source urban driving simulator CARLA (Car Learning to Act) and its publicly evaluated Leaderboard for autonomous driving performance to construct complex traffic simulation scenarios and evaluate the performance of autonomously driven vehicles trained with the proposed method.

### 4.1 Simulation environment setup and evaluation criteria

**Fig 9:**
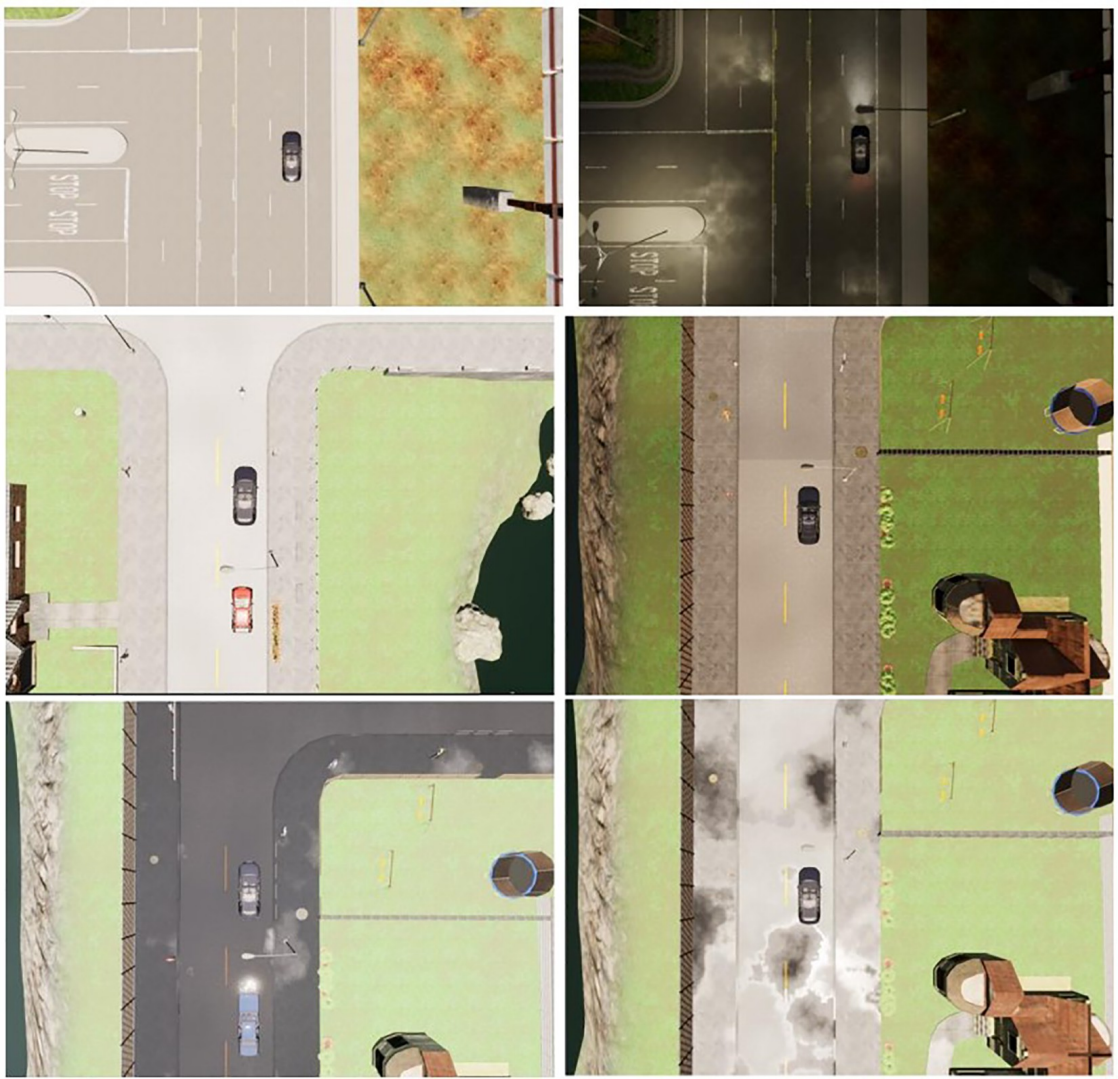
Schematic diagram of CARLA simulation environment.

Complex traffic scenarios encompass intricate road layouts and challenging road conditions. In urban autonomous driving, common complex road layouts include various intersections, roundabouts, and merge intersections, while typical complex road conditions involve obstructions (commonly referred to as "phantom obstacles"), heavy traffic, and situations where disabled vehicles obstruct the road. The combination of complex road layouts and challenging road conditions constitutes complex traffic scenarios. The CARLA-based simulation platform provides urban infrastructure, traffic signs, various weather conditions, and lighting scenarios as part of the external environment, as depicted in **Fig 9**. **Fig 9(A)** illustrates driving environments during the day (left) and night (right), while **Fig 9(B)** showcases several typical weather conditions, with the first row displaying clear and overcast days from left to right and the second row illustrating rainy and partly cloudy conditions. The hardware configuration of the computing platform and the software setup of the simulation system are detailed in **Table 1**.

**Table 1:** Hardware and simulation environment configuration.

Name	Model version
GPU	NVDIA GTX1060(6G video memory)
CPU	Inter(R) Core(TM) i7-8700
Unreal Engine	4.22.2
CARLA	0.9.10
Pytorch	1.4.0
Python	3.7.9
CUDA	10.1
cudnn	7.6
Pygame	1.9.6
Gym	0.21.0
Opencv-python	1.21.5

**Fig 10:**
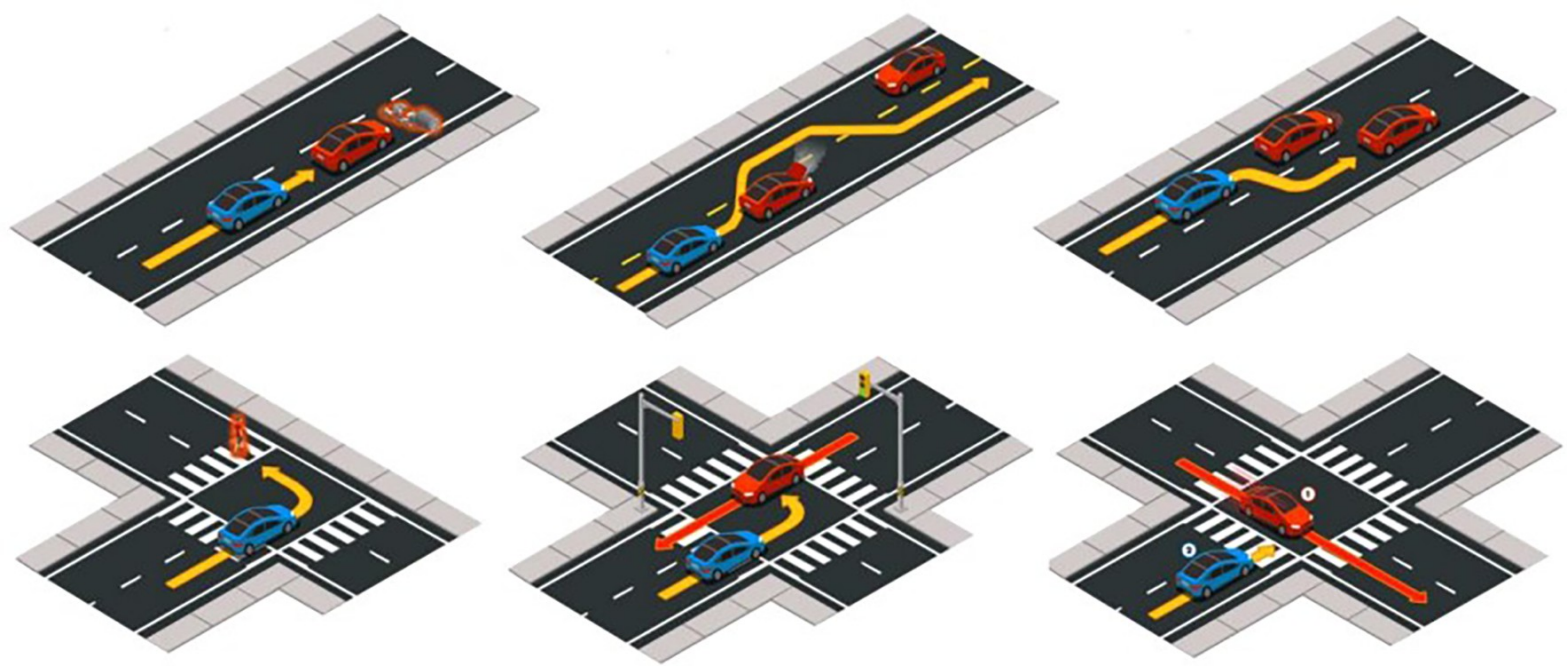
Example of a typical complex traffic scenario.

Leaderboard is CARLA’s autonomous driving ranking platform, primarily designed for assessing the driving capabilities of autonomous driving agents in real-world traffic conditions. It serves as a publicly accessible evaluation platform, offering comprehensive assessment criteria. In the tasks set on the Leaderboard, autonomous vehicles are required to navigate predefined global routes. At the starting point of each route task, the autonomous driving agent is initialized and directed to travel to a designated destination, as illustrated in Fig 10. Here, the blue point represents the starting point, the red point signifies the destination, and the green line represents the globally planned route. The established routes and tasks encompass various areas, including highways, urban settings, and residential zones. The performance of autonomous driving is evaluated under diverse weather conditions, including daytime scenarios, sunsets, rainy conditions, fog, and nighttime.

Throughout the Leaderboard testing process, autonomous vehicles encounter various types of traffic situations defined based on the criteria of the National Highway Traffic Safety Administration (NHTSA). These situations include several typical complex traffic scenarios:

1. Longitudinal planning following the lead vehicle’s abrupt braking: When the leading vehicle decelerates suddenly due to obstacles or congested road sections, the autonomous vehicle must perform emergency braking or evasive actions.
2. Evasive maneuvers in scenarios with obstructed views of suddenly emerging traffic participants: Autonomous vehicles may encounter obstructions on the road, such as buildings or roadside objects blocking their view. In such cases, they must execute emergency braking or deceleration to avoid sudden traffic participants.
3. Various interactions at intersections: While navigating through intersections, including four-way and T-shaped intersections, autonomous driving vehicles must make choices such as proceeding straight, turning left, or turning right. Negotiating interactions with various traffic participants is necessary during intersection crossings, including yielding to conflicting traffic participants or reaching compromises.
4. Lane changes to overtake slow lead vehicles: Autonomous vehicles need to change lanes to overtake slower lead vehicles, allowing them to attain higher speeds. During this process, they may also need to respond to potential conflicts in the target lane.

Fig 10 illustrates typical complex traffic scenarios encountered during everyday driving, highlighting that most complex traffic situations result from interactions between vehicles and the potential for conflicts.

### 4.2 Experimental results and analysis

All methods underwent training in the complex traffic simulation environment for 120,000-time steps. The trained models are then loaded onto autonomous vehicles in the Leaderboard test case platform configuration. Performance comparisons of different planning methods in terms of autonomous driving safety and efficiency are conducted based on the evaluation criteria outlined above. Given the distinct frameworks and types of the three methods, it is not feasible to evaluate them based solely on algorithm-specific metrics, such as reward functions. The following sections present an introduction to the relevant training and testing results, along with an analysis.

(1) Testing in a single complex traffic scenario in Town01 map

The test results in the Town01 map are presented in **Table 2**. For clarity, the test scenario involving driving at an intersection under normal traffic density conditions is presented separately.

**Table 2:** Test results at different traffic densities in Town01.

Task	Traffic density	Method	Success rate	Collision rate	Deadlock/Timeout
Straight	Sparse	DSI-LBSAC	100%	0	0
		End-to-end SAC	100%	0	0
		Morton [38]	100%	0	0
		AUTO [39]	100%	0	0
		MMFN [40]	100%	0	0
	Normal	DSI-LBSAC	98%	0	2%
		End-to-end SAC	96%	2%	2%
		Morton [38]	95%	2%	3%
		AUTO [39]	96%	2%	2%
		MMFN [40]	98%	2%	0%
	Dense	DSI-LBSAC	94%	2%	4%
		End-to-end SAC	93%	4%	3%
		Morton [38]	91%	4%	5%
		AUTO [39]	92%	4%	4%
		MMFN [40]	95%	3%	2%
Driving at intersection	Sparse	DSI-LBSAC	98%	1%	1%
		End-to-end SAC	94%	4%	2%
		Morton [38]	95%	2%	3%
		AUTO [39]	96%	2%	2%
		MMFN [40]	99%	1%	0%
	Dense	DSI-LBSAC	83%	7%	10%
		End-to-end SAC	74%	14%	12%
		Morton [38]	77%	13%	10%
		AUTO [39]	78%	10%	12%
		MMFN [40]	85%	7%	8%

Observing the results in **Table 2**, several key observations emerge: 1) In the straight driving task, the performance of various methods is remarkably close. Notably, the proposed approach exhibits a lower collision rate due to improvements in robustness and stability. As traffic density varies, success rates for all algorithms decrease, attributed to the heightened uncertainty in driving environments and increased perceptual information and observational data processing required under dense traffic conditions. 2) Navigating through intersections presents a notably greater challenge, especially in densely trafficked scenarios without coordinated traffic signals, representing a typical complex traffic scenario. The proposed method demonstrates clear advantages in handling intricate interactions in this task. Its success rate and collision rate are significantly superior to End-to-end SAC and two advanced methods, Morton and AUTO, as the proposed approach, relying on the VAE+GRU-based driving style inference network, accurately predicts the driving styles of surrounding vehicles. Furthermore, the LBSAC algorithm enhances robustness and stability, resulting in optimal safety and efficiency. A comparison with the MMFN on the CARLA Leaderboard indicates a slightly lower success rate for the proposed method, but the difference is marginal, confirming the almost identical performance and affirming the effectiveness of the proposed approach.

(2) Leaderboard testing in Town03 and Town04 maps

The trained models are tested on 20 task routes in the Town03 map and 10 task routes in the Town04 map. The results are presented in Table 3. Route completion averages are calculated according to Eq (21), where $\bar{C}o$ represents the overall average completion rate, ${\bar{c}}_{i}$ is the average completion rate for each route, and *c* is the completion level for a single test on a specific route. In Leaderboard testing, collisions do not immediately terminate a task; instead, the collision count is recorded. Consequently, a route may experience multiple collisions or none at all. Locking, leading to task timeout, occurs when autonomous vehicles are in deadlock situations. It is worth noting that some collisions may lead to deadlock, such as collisions with buildings or vegetation. The average runtime is computed as the sum of all recorded test durations divided by the total number of tests. Different methods achieve varying route completion rates, and completing more routes requires more time. Therefore, time cannot serve as the sole efficiency evaluation criterion.


$$\bar{C}o=\frac{1}{20}\sum_{i=1}^{20}{\bar{c}}_{i},{\bar{c}}_{i}=\frac{1}{10}\sum c$$ (21)

**Table 3:** Leaderboard task test results in Town03 and Town04 environments.

Map environment	Method	Average route completion degree (%)	Number of collisions	Number of deadlocks	Average running time
Town03	The proposed method	64.60%	56	32	443.59s
	End-to-end SAC	58.87%	74	34	440.98s
	Morton [38]	61.10%	65	32	442.37s
	AUTO [39]	62.74%	60	34	444.80s
	MMFN [40]	64.18%	58	32	452.64s
Town04	The proposed method	57.38%	31	41	1058.10s
	End-to-end SAC	51.70%	40	43	1029.53s
	Morton [38]	50.39%	36	43	1045.51s
	AUTO [39]	52.46%	38	43	1069.22s
	MMFN [40]	58.40%	30	39	1093.90s

According to Table 3, in comparison with advanced methods such as End-to-end SAC, Morton, AUTO, and MMFN, the proposed approach achieves the highest completion rate, the fewest collisions, and the lowest instances of deadlock. Additionally, the proposed method exhibits lower runtimes compared to these methods, indicating higher driving efficiency and robustly affirming its effectiveness. Furthermore, since Town04 has a larger map and longer routes, its runtime is significantly higher than in the Town03 environment. Thus, from the perspective of autonomous driving planning alone, the performance of the DSI-LBSAC method in comprehensive aspects like planning safety (few collisions), efficiency, and availability clearly outshines traditional end-to-end planning and imitation learning methods. A comparison with MMFNon the CARLA Leaderboard reveals that on Town03, the proposed method has a slightly superior completion rate and fewer collisions than MMFN. However, on Town04, the proposed method has a slightly lower completion rate and more collisions than MMFN. Overall, the proposed method and MMFN exhibit distinct strengths, and their performances are essentially equivalent.

In the Town03 test cases, the proposed method excels by achieving 100% route completion in several routes: route5, route7, route8, route14, route15, and route17. However, in some route tasks (e.g., route4, 9, 10), vehicles trained by all methods exhibit lane departures and fail to complete even 10% of the entire route. This issue may be attributed to path redundancy in these routes. The same problem is observed in the Town04 map.

**Fig 11:**
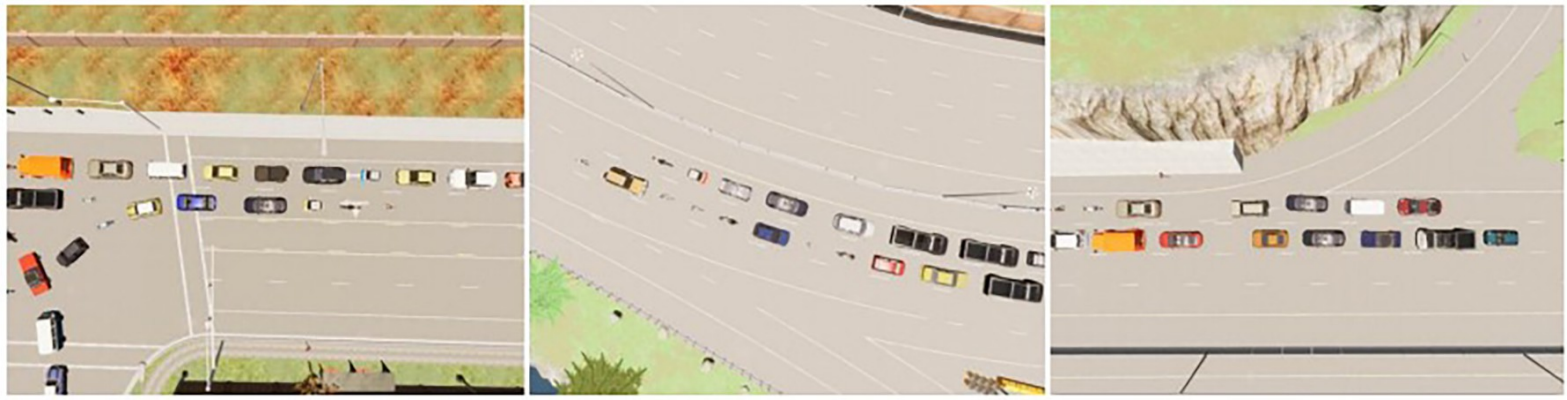
Deadlock scenarios during testing.

Two main factors contribute to deadlocks (timeouts): deadlocks resulting from collisions and congestion-induced road segment deadlocks. The former occurs when autonomous vehicles collide with roadside objects or are pushed off the road by other vehicles. Since reversing is not part of the research and experiments, they remain trapped without escape. Visualizing the driving process in sections where deadlocks consistently occur reveals the root causes, as illustrated in **Fig 11**. In these scenarios, autonomous vehicles become ensnared in traffic, and due to spatial constraints, they cannot execute lane-changing maneuvers as a regular vehicle might. Even human drivers would face challenges in such extreme traffic conditions, necessitating lane-crossing over solid lines or guidance from traffic officers. However, current autonomous driving systems are designed to adhere strictly to traffic regulations to ensure safety. Therefore, when trapped, they deadlock and eventually time out, making it impossible to complete the route. This situation is more prevalent in the Town04 map.

### 4.3 Sensitivity analysis

The proposed method comprises an overarching framework that includes a driving style prediction inference network based on VAE and GRU, as well as improvements to the LBSAC deep reinforcement learning algorithm within an Actor-Critic framework. To enhance the performance of the LBSAC algorithm, an attention module is integrated. To ensure its safety in uncertain environments, a safety layer is implemented.

To validate the effectiveness of the driving style inference network, the attention module, and the safety layer in practical applications, three ablation models are trained in the Town03 map environment: models without the inference module, without the attention module (No attention, No-attn), and without the safety layer (No safety threshold, No-st). Six routes, namely route5, route7, route8, route14, route15, and route17, which performed well in the previous tests, are selected for evaluation. Each ablation model underwent ten tests per route, and the results are compared against the DSI-LBSAC algorithm’s test outcomes.

According to **Table 4**, the following conclusions can be drawn:(1) The driving style inference module significantly enhances the planning module’s performance. All models incorporating the driving style inference network outperform models using only the LBSAC algorithm. By predicting the driving styles of surrounding vehicles, the driving style network effectively avoids potential conflicts, thus preventing situations that may lead to deadlocks. (2) The attention module has a noticeable impact on the efficiency of the planning methods. Models that lack the attention module exhibit the longest average runtimes, with an additional time requirement of 16.92 to 50.92 seconds compared to other models. This is particularly evident when the completion rates of models without the attention mechanism and the original model surpass those of models without the safety layer. Even in cases where the attention-free model’s completion rate is higher, its runtime remains relatively high. The attention module assigns varying weights to vehicles with distinct features and positions, allowing it to focus on vehicles that have a greater impact on autonomous driving. Especially in complex traffic scenarios, the attention module enhances the efficiency and reduces collision rates, affirming the necessity of this mechanism. (3) The safety layer plays a crucial role in the safety of autonomous driving planning methods. Models without the safety layer experience significantly more collisions than other models. Even with the inclusion of the attention module, it cannot guarantee the safety of planning output strategies in highly uncertain and complex scenarios. Through testing records, it is observed that models without the safety layer perform poorly when dealing with unexpected situations, particularly in scenarios where pedestrians suddenly emerge from obscured areas, such as open environments. These experiments confirm the safety layer’s effectiveness in handling planning safety in uncertain environments.

**Table 4:** Comparison of ablation model test results.

Map environment	Method	Average route completion degree (%)	Number of collisions	Number of deadlocks	Average running time
Town03	The proposed method	100%	6	0	542.56s
	LBSAC	98.37%	11	2	576.56s
	No-attn	99.04%	12	0	593.48s
	No-st	99.65%	17	0	558.32s

### 4.4 Generalization performance evaluation

**Fig 12:**
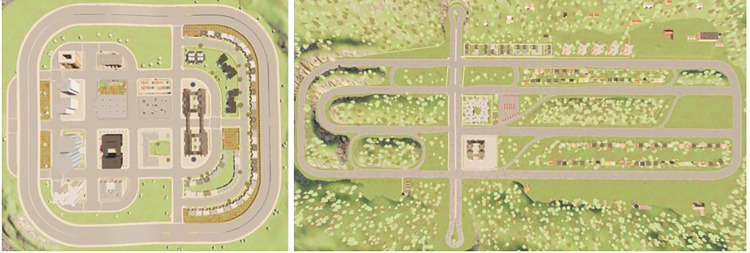
Top view of Town05 (left) and Town06 (right).

To assess the algorithm’s generalization and transferability, the proposed models are tested on new maps, Town05 and Town06, using the task routes provided by Leaderboard. Leaderboard offers ten distinct routes for both Town05 and Town06, and constructs complex traffic scenarios during the driving process to evaluate autonomous vehicles. Town05 and Town06 are depicted in **Fig 12**. Town05 is a multi-lane urban map with numerous intersections, while Town06 is classified as a highway map.

To assess the algorithm’s generalization, models trained in corresponding maps (e.g., Town05 and Town03) and (e.g., Town06 and Town04) are directly tested in the new maps. This validation approach ensures the generalization of the method. The test results are presented in **Table 5**.

**Table 5:** Results of model adaptation tests.

Map environment	Average route completion degree (%)	Number of collisions	Number of deadlocks
Town05	63.89%	49	18
Town06	58.67%	30	32

When comparing the model’s performance in new maps to the results presented in Table 3, we observed a slight decrease of 0.71% in completion rate and a gain of 1.29% in completion rate for Town05 and Town06, respectively. Overall, the performance variations are minimal. Despite the simplicity of routes in Town05 and Town06 compared to Town03 and Town04, the proposed model exhibited improvements in metrics such as collision rates. When transferred to new maps, the model maintained its safety, stability, and overall performance. This generalization experiment confirms the method’s robust generalization and transferability.

## 5. Conclusions

In response to the interactions among vehicles and the potential impact of driving styles on autonomous driving path planning, along with the limited robustness of existing deep reinforcement learning methods, we introduced a robust deep reinforcement learning approach aided by driving styles. This approach aims to train autonomous vehicles to learn motion planning strategies. By combining a driving style inference network based on VAE+GRU and an improved deep reinforcement learning method, we achieved safe interactions between autonomous vehicles and surrounding vehicles in complex traffic scenarios. We explored the possibilities of closely integrating predictive reasoning modules with deep reinforcement learning-based planning methods.

A complex traffic simulation environment is created using the CARLA simulator and the Leaderboard platform. Experiments are conducted under varying traffic densities and distributions of driving-style vehicles to demonstrate the effectiveness of the proposed planning approach in enabling autonomous vehicles to safely interact with participants exhibiting different driving styles.

While the method presented in this paper effectively addresses the challenges posed by certain complex traffic scenarios and facilitates safe interactions between autonomous vehicles and other vehicles, there are further exploratory paths to consider in practical applications. Enhancing the extension of the proposed inference method to infer higher-level latent states like driving intent and combining more advanced predictions, such as trajectory prediction, with deep reinforcement learning-based planning methods, is an avenue for future research.

## References

[1] LIN Y S U N K, Bashir A K. KeyLight: Intelligent Traffic Signal Control Method Based on Improved Graph Neural Network[J]. IEEE Transactions on Consumer Electronics, 2023.

[2] Early Estimates of Motor Vehicle Traffic Fatalities and Fatality Rate by Sub-Categories in 2020 [R]. USA: NHTSA, 2021:1–10.

[3] Dannenberg A L, Rodriguez D A, Sandt L S. Advancing research in transportation and public health: A selection of twenty project ideas from a US research roadmap[J]. Journal of Transport & Health, 2021, 21: 101021.

[4] Cho R L T, Liu J S, Ho M H C. The development of autonomous driving technology: perspectives from patent citation analysis[J]. Transport Reviews, 2021, 41(5): 685–711.

[5] Liu L, Lu S, Zhong R, et al. Computing systems for autonomous driving: State of the art and challenges[J]. IEEE Internet of Things Journal, 2020, 8(8): 6469–6486.

[6] Chen L, Li Y, Huang C, et al. Milestones in autonomous driving and intelligent vehicles: Survey of surveys[J]. IEEE Transactions on Intelligent Vehicles, 2022, 8(2): 1046–1056.

[7] Cheng J, Zhang L, Chen Q, et al. A review of visual SLAM methods for autonomous driving vehicles[J]. Engineering Applications of Artificial Intelligence, 2022, 114: 104992.

[8] Singandhupe A, La H M. A review of slam techniques and security in autonomous driving[C]//2019 third IEEE international conference on robotic computing (IRC). IEEE, 2019: 602–607.

[9] Naumann M, Konigshof H, Lauer M, et al. Safe but not overcautious motion planning under occlusions and limited sensor range[C]// IEEE Intelligent Vehicles Symposium (IV). IEEE, 2019:140–145.

[10] Wang Z, Liao X, Wang C, et al. Driver behavior modeling using game engine and real vehicle: A learning-based approach[J]. IEEE Transactions on Intelligent Vehicles, 2020, 5(4): 738–749.

[11] Azadani M N, Boukerche A. Driving behavior analysis guidelines for intelligent transportation systems[J]. IEEE transactions on intelligent transportation systems, 2021, 23(7): 6027–6045.

[12] Yurtsever E, Lambert J, Carballo A, et al. A survey of autonomous driving: Common practices and emerging technologies[J]. IEEE access, 2020, 8: 58443–58469.

[13] Kou Y, Ma C. Dual-objective intelligent vehicle lane changing trajectory planning based on polynomial optimization[J]. Physica A: Statistical Mechanics and its Applications, 2023, 617: 128665.

[14] Chen R, Hu J, Xu W. An RRT-Dijkstra-Based Path Planning Strategy for Autonomous Vehicles[J]. Applied Sciences, 2022, 12(23): 11982.

[15] Yu C, Ni A, Luo J, et al. A novel dynamic lane-changing trajectory planning model for automated vehicles based on reinforcement learning[J]. Journal of advanced transportation, 2022, 2022.

[16] Yang C, Chen X, Lin X, et al. Coordinated trajectory planning for lane-changing in the weaving areas of dedicated lanes for connected and automated vehicles[J]. Transportation Research Part C: Emerging Technologies, 2022, 144: 103864.

[17] Yu Y, Luo X, Su Q, et al. A dynamic lane-changing decision and trajectory planning model of autonomous vehicles under mixed autonomous vehicle and human-driven vehicle environment[J]. Physica A: Statistical Mechanics and its Applications, 2023, 609: 128361.

[18] Wu J, Chen X, Bie Y, et al. A co-evolutionary lane-changing trajectory planning method for automated vehicles based on the instantaneous risk identification[J]. Accident Analysis & Prevention, 2023, 180: 106907. 36455450 10.1016/j.aap.2022.106907

[19] Qiao Z, Muelling K, Dolan J, et al. POMDP and hierarchical options MDP with continuous actions for autonomous driving at intersections[C]// International Conference on Intelligent Transportation Systems. IEEE, 2018: 2377–2382.

[20] Ding W, Zhang L, Chen J, et al. Safe Trajectory generation for complex urban environments using spatio-temporal semantic corridor[J]. IEEE Robotics and Automation Letters, 2019, 4(3):2997–3004.

[21] Zhang Lu, et al. Efficient uncertainty-aware decision-making for automated driving using guided branching[C]// IEEE International Conference on Robotics and Automation. IEEE, 2020: 3291–3297.

[22] Li X, Guvenc L, Aksun-Guvenc B. Decision Making for Autonomous Vehicles[J]. arXiv preprint arXiv:2304.13908, 2023.

[23] Li T, Zhang L, Liu S, et al. MARC: Multipolicy and Risk-aware Contingency Planning for Autonomous Driving[J]. IEEE Robotics and Automation Letters, 2023.

[24] Fernando T, Denman S, Sridharan S, et al. Deep inverse reinforcement learning for behavior prediction in autonomous driving: Accurate forecasts of vehicle motion[J]. IEEE Signal Processing Magazine, 2020, 38(1): 87–96.

[25] Zhang L, Zhang R, Wu T, et al. Safe reinforcement learning with stability guarantee for motion planning of autonomous vehicles[J]. IEEE Transactions on Neural Networks and Learning Systems, 2021, 32(12): 5435–5444. 34242172 10.1109/TNNLS.2021.3084685

[26] Moghadam M, Alizadeh A, Tekin E, et al. An End-to-end deep reinforcement learning approach for the long-term short-term planning on the frenet space[J]. 2020. arXiv preprint arXiv:2011.13098

[27] Li S, Wei C, Wang Y. Combining decision making and trajectory planning for lane changing using deep reinforcement learning[J]. IEEE Transactions on Intelligent Transportation Systems, 2022, 23(9): 16110–16136.

[28] Gu S, Chen G, Zhang L, et al. Constrained reinforcement learning for vehicle motion planning with topological reachability analysis[J]. Robotics, 2022, 11(4): 81.

[29] Hu H, Yang X, Xiao S, et al. Anti-conflict AGV path planning in automated container terminals based on multi-agent reinforcement learning[J]. International Journal of Production Research, 2023, 61(1): 65–80.

[30] Ma X, Li J, Kochenderfer M J, et al. Reinforcement learning for autonomous driving with latent state inference and spatial-temporal relationships[C]//2021 IEEE International Conference on Robotics and Automation (ICRA).IEEE, 2021: 6064–6071.

[31] Chakraborty N, Hasan A, Liu S, et al. Structural Attention-Based Recurrent Variational Autoencoder for Highway Vehicle Anomaly Detection[J]. arXiv preprint arXiv:2301.03634, 2023.

[32] Arbabi S, Tavernini D, Fallah S, et al. Learning an interpretable model for driver behavior prediction with inductive biases[C]//2022 IEEE/RSJ International Conference on Intelligent Robots and Systems (IROS). IEEE, 2022: 3940–3947.

[33] Liu S, Chang P, Chen H, et al. Learning to Navigate Intersections with Unsupervised Driver Trait Inference[C]//2022 International Conference on Robotics and Automation (ICRA). IEEE, 2022: 3576–3582.

[34] Wang X, Tang K, Dai X, et al. Safety-balanced driving-style aware trajectory planning in intersection scenarios with uncertain environment[J]. IEEE Transactions on Intelligent Vehicles, 2023.

[35] Liu X, Wang Y, Jiang K, et al. Interactive trajectory prediction using a driving risk map-integrated deep learning method for surrounding vehicles on highways[J]. IEEE Transactions on Intelligent Transportation Systems, 2022, 23(10): 19076–19087.

[36] Albeaik S, Bayen A, Chiri M T, et al. Limitations and improvements of the intelligent driver model (IDM)[J]. 2021. arXiv preprint arXiv:2104.02583. doi: 10.1109/IROS47612.2022.9981775

[37] Zhao K, Liu Z, Zhao B, et al. Class-Aware Adversarial Multiwavelet Convolutional Neural Network for Cross-Domain Fault Diagnosis[J]. IEEE Transactions on Industrial Informatics, 2023.

[38] Morton J, Kochenderfer M. Simultaneous policy learning and latent state inference for imitating driver behavior[J]. 2017. arXiv preprint arXiv:1704.05566

[39] Chen D., Zhou B., Koltun V., and Krihenbiihl P., Learning by cheating, in Conference on Robot Learning, 2020, pp. 66–75.

[40] Q. Zhang, M. Tang, R. Geng, F. Chen, R. Xin and L. Wang, "MMFN: Multi-Modal-Fusion-Net for End-to-End Driving," 2022 IEEE/RSJ International Conference on Intelligent Robots and Systems (IROS), Kyoto, Japan, 2022, pp. 8638–8643, doi: 10.1109/IROS47612.2022.9981775

